# Comprehensive immune profiling of dengue and chikungunya viral responses using a novel miniaturized automated whole blood cellular analysis system and mass cytometry in a pediatric cohort in Msambweni, Kenya

**DOI:** 10.1093/immhor/vlaf006

**Published:** 2025-03-06

**Authors:** Sangeeta Kowli, Amy Krystosik, Matthew Hale, Francis Mutuku, Jael S Amugongo, Said L Malumbo, Phillip K Chebii, Priscillah W Maina, Kavita Mathi, Elysse N Grossi-Soyster, Mary Rieck, Angelle Desiree LaBeaud, Holden T Maecker

**Affiliations:** Institute for Immunity, Transplantation, and Infection, Stanford University School of Medicine, Stanford, CA, United States; Department of Pediatrics, Division of Infectious Disease, Stanford University School of Medicine, Stanford, CA, United States; Smart Tube Inc, Menlo Park, CA, United States; Department of Environment and Health Sciences, Technical University of Mombasa, Mombasa, Kenya; Department of Environment and Health Sciences, Technical University of Mombasa, Mombasa, Kenya; Vector-Borne Diseases Unit, Msambweni County Referral Hospital, Msambweni, Kwale, Kenya; Vector-Borne Diseases Unit, Msambweni County Referral Hospital, Msambweni, Kwale, Kenya; Vector-Borne Diseases Unit, Msambweni County Referral Hospital, Msambweni, Kwale, Kenya; Vector-Borne Diseases Unit, Msambweni County Referral Hospital, Msambweni, Kwale, Kenya; Institute for Immunity, Transplantation, and Infection, Stanford University School of Medicine, Stanford, CA, United States; Department of Pediatrics, Division of Infectious Disease, Stanford University School of Medicine, Stanford, CA, United States; Institute for Immunity, Transplantation, and Infection, Stanford University School of Medicine, Stanford, CA, United States; Department of Pediatrics, Division of Infectious Disease, Stanford University School of Medicine, Stanford, CA, United States; Institute for Immunity, Transplantation, and Infection, Stanford University School of Medicine, Stanford, CA, United States

**Keywords:** Chikungunya (CHIKV), Dengue (DENV), CyTOF, fixed whole blood processing, pediatric immunology

## Abstract

Chikungunya (CHIKV) and dengue (DENV) are mosquito-borne viruses that cause severe epidemics, often in remote regions. A limitation to our understanding of these pathogens is the difficulty of performing assays of the cellular immune response. To fill this gap, we developed a novel miniaturized automated system capable of processing 250 μl of whole blood for high-throughput cellular analysis. In a field study with a pediatric cohort in Msambweni, Kenya, known for previous exposure to CHIKV and/or DENV, we processed 133 whole blood samples using our system under three conditions: no stimulation, and stimulation with CHIKV or DENV peptide pools. These samples underwent CyTOF or flow cytometry analysis to evaluate virus-specific memory T cell responses and phenotypes. CyTOF analysis of 81 participant samples revealed significant cytokine responses to CHIKV and DENV, particularly IFNγ (*P* < 0.01 and *P* < 0.0001, respectively) and TNF-α (*P* < 0.0001) by γδ T cells. Additionally, a significant TNF-α response was observed in the CD8+ TEMRA memory subset to DENV, albeit to a lesser degree than in γδ T cells. To confirm our CyTOF findings, we employed flow cytometry on the remaining 40 samples using a targeted panel, validating significant TNF-α (*P* < 0.0001 and *P* < 0.01) and IFN-γ (*P* < 0.05) responses by γδ T cells to CHIKV and DENV, respectively. Our study demonstrates that our innovative automated system enables detailed assessment of immune function, particularly beneficial in pediatric populations and resource-limited settings with limited sample volumes. This approach holds promise for advancing our understanding of cellular immune responses to various viral and infectious diseases.

## Introduction

Recent years have witnessed a rapid emergence of arbovirus diseases, such as those caused by chikungunya virus (CHIKV) and dengue virus (DENV).^1^^,^^2^  *Aedes aegypti* is the principal vector responsible for the transmission of both CHIKV and DENV.^3^^,^^4^ CHIKV is an alphavirus of the *Togaviridae* family and has spread in Africa, Europe, Asia and to several regions in America since its first reported case in 2013.^5^ CHIKV frequently causes chronic impairments such as encephalitis and debilitating arthritis that can affect the quality of life of the infected individuals.^1^ DENV belongs to the *flavivirus* genus of the *Flaviviridae* family, and the geographical expansion of DENV viral incidences includes Africa, South-East Asia, Eastern Mediterranean and America. Clinical manifestations of DENV infection can range from mild fever to life-threatening hemorrhagic fever.^6–8^ Further, treatment options for both CHIKV and DENV infections are mostly supportive, creating an urgent need to deepen our understanding on the pathogenesis of these viral infections to aid both in the development of effective vaccines and treatment.^9^

In Kenya, there were recent outbreaks of CHIKV reported in Mandera in 2016, and DENV in Mombasa in 2013 and 2017;^4^ however, the lack of testing facilities in Kenya and other developing nations that can provide a comprehensive analysis of the viral responses has been a major limitation in the field. We, therefore, decided to evaluate the immune profile by high-throughput technology mass cytometry (CyTOF) in an ongoing pediatric cohort in Msambweni, Kenya with exposure to both DENV and CHIKV.^10–18^ CyTOF is a high-parameter technology that permits the detection of ∼40 phenotypic and/or functional immune markers simultaneously. This allowed us to do an in-depth analysis of the viral immune responses of children in Msambweni exposed to DENV and CHIKV at both the phenotypic and functional levels characterized by the expression of cytokines, cytotoxic markers, and activation markers.

To simplify cellular analysis in field settings, Smart Tube, Inc. (USA), developed a novel miniaturized automated system for initial whole blood processing that included stimulation, lysis, and fixation. The fixed whole blood samples could then be stored at −80°C and later processed for testing by CyTOF. The benefits of this novel system were manifold. First, the compact design allowed for easy transportation of the system to the field study site in Msambweni, Kenya. Second, the system required very small volumes of whole blood as input (250 μl per stimulation condition, 1 ml in total), overcoming a potential drawback of obtaining large volumes of blood especially in pediatric populations. Finally, the automated design of the sample-sparing prototype also eliminated the need for hands-on intervention.

## Materials and methods

### Cohort

This substudy recruited participants from two larger cohorts: an acutely ill cohort (previously described in^11^^,^^13^^,^^16^ and a longitudinal healthy community cohort.^10^ Both cohorts were part of a larger arbovirus study (NIH R01AI102918, SSC No. 2611) conducted in Kenya.

### Substudy eligibility criteria

To be eligible for inclusion in this substudy, participants had to meet the following criteria: (1) Have at least 2 data points including ELISA testing to determine CHIKV/DENV seroconversion status; (2) Be aged 1 - <18 years (3); Have been enrolled in either the acutely ill cohort or the longitudinal healthy community cohort in either the Ukunda or Msambweni study site; and (4) Have consented to be included in follow-up studies.

This comprehensive approach allowed for a detailed analysis of CHIKV/DENV seroconversion patterns in both symptomatic and asymptomatic pediatric populations across diverse geographical and demographic settings in Kenya.

**Acutely Ill Cohort:** Participants were recruited from January 2014 to August 2018 at 4 study areas: Chulaimbo, Kisumu, Msambweni, and Ukunda. The study sites included 5 health facilities: Chulaimbo County Hospital and Mbaka Oromo Dispensary in Chulaimbo, Jaramogi Oginga Odinga Teaching and Referral Hospital (including Obama Children's Hospital) in Kisumu, Msambweni County Hospital in Msambweni, and Diani Health Centre in Ukunda. These sites were selected to provide geographical diversity, representing both western (Lake Victoria region) and coastal Kenya, as well as rural and urban settings.

**Inclusion criteria for the acutely ill cohort were: (**1) children aged 1 to <18 years; (2) presenting with acute febrile illness (temperature ≥38°C or reported fever); (3) no localizing signs or symptoms; and (4) meeting criteria for possible arboviral infection. Children with localizing illnesses (eg traumatic injury, acute pneumonia, urinary tract infections) were excluded.

**Longitudinal Healthy Community Cohort:** This cohort followed children aged 1 to 12 years at enrollment from January 2014 to December 2018. Participants were recruited house-to-house in both densely and sparsely populated areas of Western Kenya (Kisumu, Chulaimbo) and Coastal Kenya (Ukunda, Msambweni).

### Study procedures

For both cohorts, written informed consent was obtained from parents/guardians, with verbal assent from children ≥7 years. Certified clinical officers conducted detailed clinical histories and physical examinations. Blood samples were collected by phlebotomy for malaria testing (microscopy or RDT) and arbovirus testing (DENV and CHIKV IgG ELISA).

The acutely ill cohort participants were asked to return for a follow-up visit after 4 weeks. The longitudinal cohort was followed for up to 5 years with approximately 6-month interval visits.

Data collection included demographic information, socioeconomic indicators, mosquito exposure, travel history, GPS data, and household factors. Height and weight measurements were taken to calculate growth and nutritional status indicators. The PedsQL™ 4.0 SF15 health-related quality of life survey was administered at initial and follow-up visits for the acutely ill cohort.

### Data management

Data were collected using Open Data Kit (ODK) and REDCap and stored securely in REDCap.

### Sample collection

**Figure 1.**
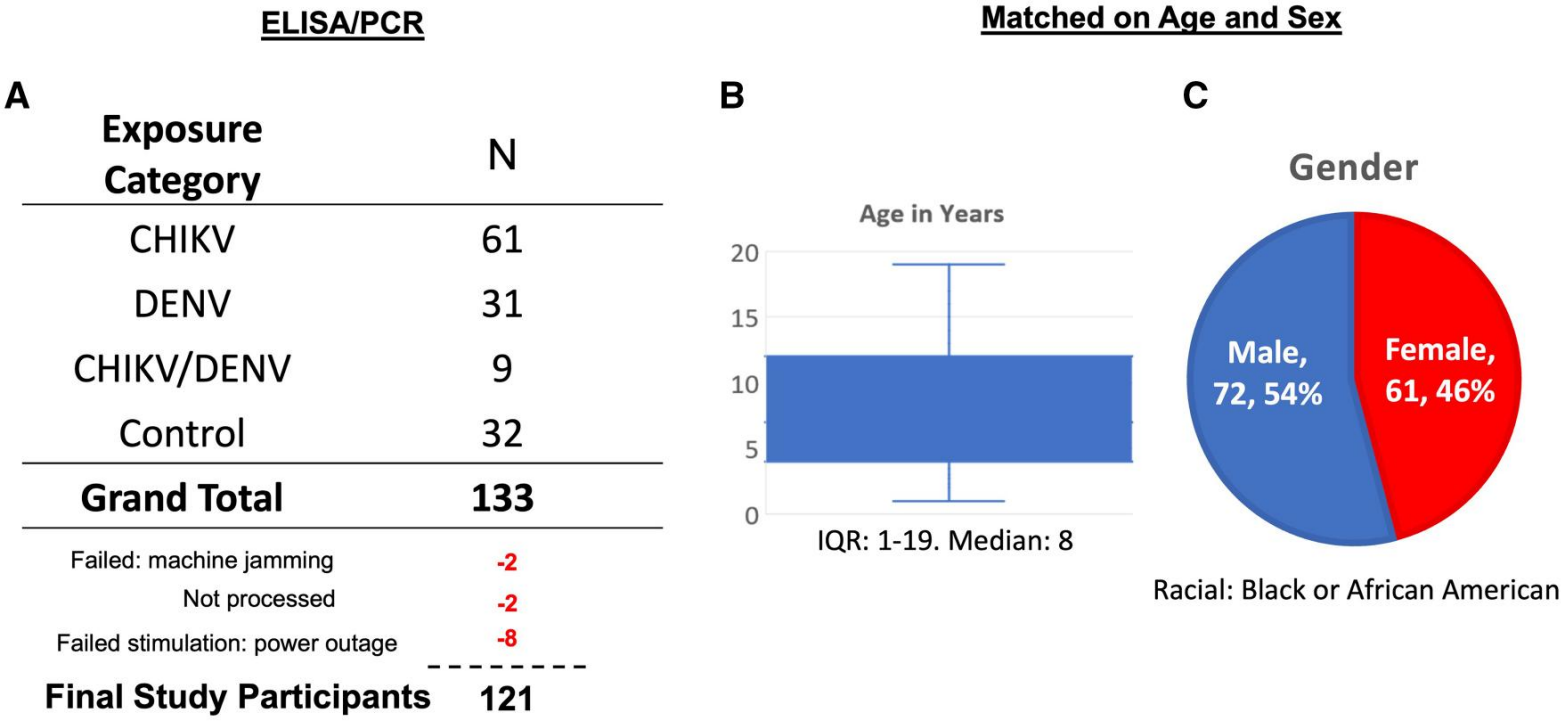
Exposure and demographic variables of the study participants. Exposure to chikungunya and/or dengue virus was assessed using ELISA. Of the 133 samples collected, 2 were excluded due to machine issues, such as jamming. Additionally, 2 samples were not processed because of machine unavailability, and eight samples were excluded due to failed stimulations caused by a power outage at the facility. Consequently, 121 cartridges were classified as “good” samples for downstream analysis, representing a high success rate for a prototype instrument (A). The median age of participants was 8 years (B), and the cohort consisted of a slightly higher percentage of males (54%) compared to females (C).

In March 2018, whole blood samples from 133 child participants were obtained with parental consent under a protocol approved through the Technical University of Mombasa. Samples were processed using the Smart Tube automated cartridge system for three conditions: no stimulation (unstim), and stimulation with CHIKV or DENV peptide pools. Each cartridge corresponded to samples from a single participant for the three conditions. The frozen cartridges were then shipped to Stanford, California, USA for further testing. Samples from 121 to 133 cartridges were tested for viral responses by CyTOF or flow cytometry. Twelve cartridges were excluded from the study as sample processing was compromised due to technical challenges (Fig. 1A). Exposure status to CHIKV and DENV virus as characterized by IgG response to CHIKV and/or DENV as measured by ELISA^14^ and demographics for this study cohort are presented in Fig. 1A–C. The study was conducted under the supervision of the institutional review board of Stanford University (Stanford, California, USA; IRB-38677) and the scientific and ethics review unit of the Kenya Medical Research Institute (Kisumu, Kenya; SSC 2611).

Samples were tested for DENV and CHIKV exposure using a validated in-house ELISA protocol to detect anti-DENV and anti-CHIKV IgG, as previously described (https://pubmed.ncbi.nlm.nih.gov/29040262/). Briefly, DENV and CHIKV (using vaccine strain 181/25) viral antigens were produced in vitro in Vero cells and stored at −80°C. The ELISA protocol was three days long. On day one, Nunc-immuno flat bottom 96 well plates were coated with 50ul of the diluted (with 1X PBS) viral antigens (final concentration 25 ug/ml) and incubated overnight at 4°C. On day two, the plates were taken out of incubation, washed, loaded with a blocking buffer, and incubated at 37°C for 2 hours. In the meantime, sera were diluted with the blocking buffer with a 1:100 ratio. After 2 hours of incubation, the blocking buffer was discarded, the plates were washed, and 50 ul of sera was loaded in each well along with positive, negative, and blank controls. The plates were then incubated overnight at 4°C again. On day 3, the plates were taken out of 4°C and incubated at 37°C for one hour. After the one-hour incubation, the plates were washed and loaded with 50 ul of secondary antibody. For the secondary antibody, an Alkaline Phosphatase-conjugated AffiniPure Goat Anti-Human IgG Fcγ Fragment from Jackson ImmunoResearch Laboratories diluted in blocking buffer 1:2000 was used. The plates loaded with secondary antibody were incubated again at 37°C for an hour. After incubation, the plates were washed again and loaded with 100ul of PNPP tablets dissolved in PNPP solution from ThermoFisher Scientific to detect alkaline phosphatase. After incubating the plates for 30 minutes at 37°C, the plates were read at 405 nm absorbance. Pooled sera previously confirmed by plaque reduction neutralization test (PRNT) were used as positive and negative controls and to determine cutoff values for the detection of anti-DENV and anti-CHIKV IgG antibodies from the sera being tested.

### DENV and CHIKV peptide selection

An equimolar mega pool mix of 268 peptides (9 to 15 amino acids for each peptide) corresponding to the known epitopes of DENV were obtained from Dr. Alessandro Sette and Dr. Daniela Weiskopf at La Jolla Institute for Immunology. For CHIKV, we tested several overlapping 15-mer peptides that included E1, E2, STR, NSP2, and NSP3. To determine the specific CHIKV antigen for stimulation, we performed flow cytometry. Briefly, PBMCs from 13 subjects who were exposed to CHIKV and/or DENV were tested for their response to the various CHIKV peptides (PBMCs were provided by Dr William Messer, OHSU). PBMCs from the 13 subjects were split into the following conditions in order of priority of testing: unstimulated, NSP2, NSP3, E2, E1, STR, and PMA/ionomycin stimulation. Cells were rested overnight at 37°C, 5% CO_2._ The next day, protein inhibitors brefeldin A and monensin were added to all the cells and cells were either left unstimulated (6 h) or stimulated with the various CHIKV antigens (6 h) or PMA/ionomycin (4 h) at 37°C, 5% CO_2._ After stimulation, cells were labeled with a PE Texas Red cell viability dye for 20 min at RT, following which cells were washed and then incubated for 30 min in the dark at RT for surface markers CD3 (BV510), CD4 (PerCPCy 5.5) and CD8 (BV421) T cells. Post surface labeling, cells were washed, fixed with 2% PFA for 10 min at RT, permeabilized and intracellular antibodies were added for markers IFN-γ (FITC), TNF-α (Alexa Fluor 700), IL-2 (PE-Cy7), IL-4 (APC), and IL-17 (PE) in the dark for 1 h on ice. Finally, cells were washed and acquired by flow cytometry. Our analysis showed CD4^+^ IFN-γ responses to be the strongest compared to the other cytokines. The NSP2 CHIKV peptide pool showed the strongest CD4^+^ IFN-γ responses compared to the other peptide pools and therefore this peptide pool was selected for the field study (Fig. S1).

### Selection of lyosphere formulation for protein inhibitors brefeldin a and monensin

Three different lyosphere formulations, B144, B346 and B407, manufactured by BIOLYPH_LLC_ (MN, USA) were tested by CyTOF. Briefly, PBMCs from a healthy donor were thawed and split into unstimulated and PMA/ionomycin stimulated conditions and rested overnight at 37°C, 5% CO_2_. The next day, CD107a (Standard BioTools, 1:100 dilution as per manufacturer’s recommendation) was added to all the wells and PBMCs from both conditions were treated with either brefeldin A (5 μg/ml) or monensin (5 μg/ml) lyospheres or a combination of both brefeldin A + Monensin (50 ng/ml) lyospheres from all 3 excipient formulations. Cells were also treated with brefeldin A + monensin (50 ng/ml) in TBA and brefeldin A + monensin (50 ng/ml) in PBS for comparative purposes. Cells were incubated for 4 h at 37°C, 5% CO_2_. At the end of the stimulations, cells were treated with EDTA for 15 min at RT, following which cells were washed, and antibodies for surface markers were added using previously determined titres. Cells were then incubated with 115-DOTA malemide for live/dead cells and incubated for 30 min on ice, following which cells were washed and fixed in 2% paraformaldehyde (PFA) overnight at 4°C. The next day, cells were washed, permeabilized and antibodies for intracellular markers were added using previously determined titres. After this, cells were washed in CyFACS followed by DNA staining for 20 min at RT. Finally, cells were first washed in CyFACS and then milliQ water following which cells were acquired on CyTOF by diluting in EQ^TM^ Calibration beads (Standard BioTools, Markam, CA). We examined the effects of each formulation on the CD4+ and CD8+ T cell cytokine expression (IFN-γ, TNF-α, IL-2, IL-4, and IL-17). We wanted an excipient formulation that had the lowest biological effect, and we found the B407 excipient with the combined Brefeldin A + Monensin lyosphere showed the lowest background, while preserving cytokine responses, compared to both the B144 and B346 excipient formulation (Fig. S2).

### Automated stimulation and fixation

**Figure 2.**
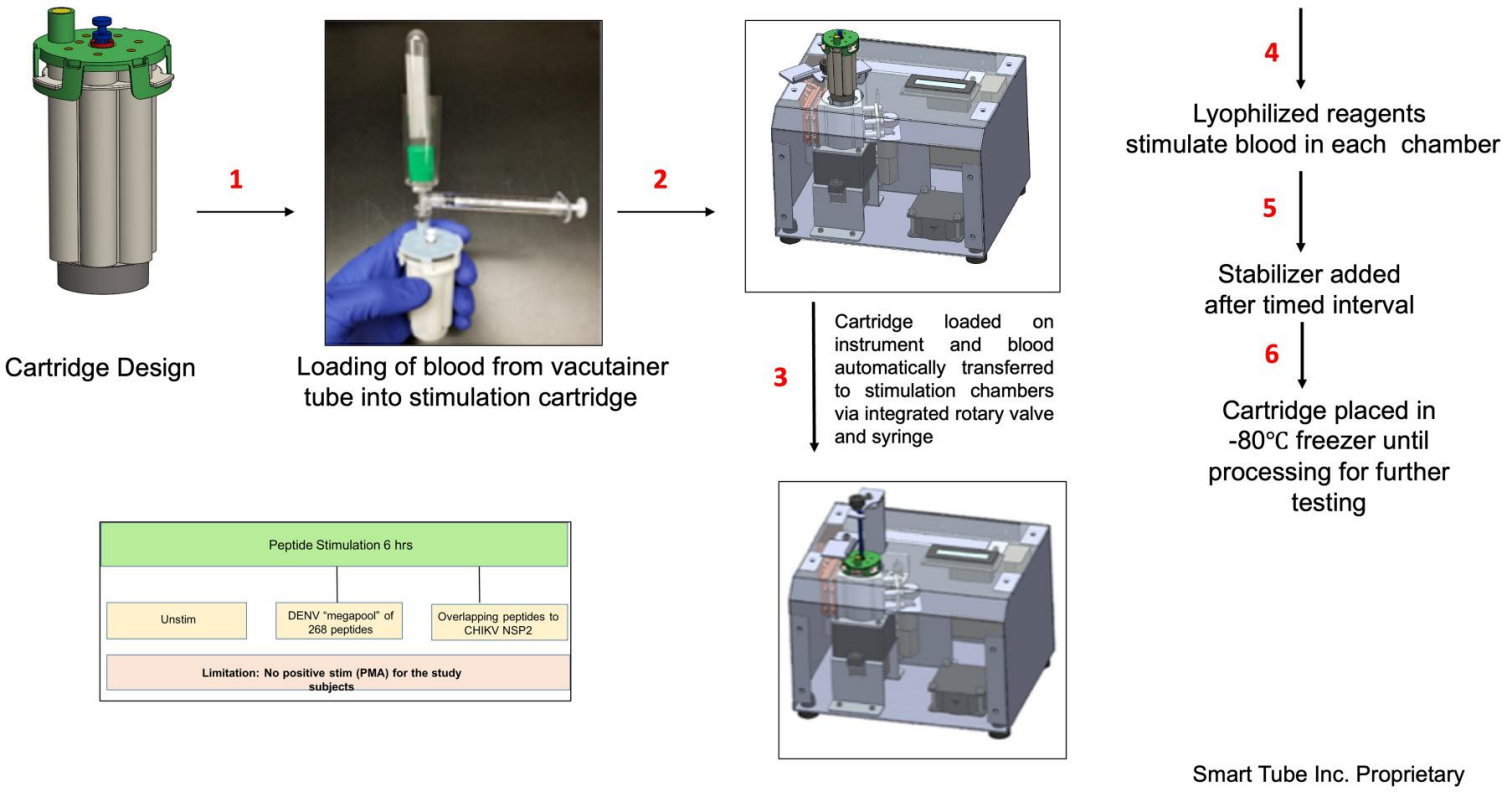
Automated stimulation and fixation of whole blood samples by the cartridge system. Whole blood was loaded onto the cartridge, where it was automatically aliquoted into 3 incubation chambers for stimulation with chikungunya or dengue peptide pools or left unstimulated. After a timed 6-hour incubation at 37°C, the samples were automatically stabilized by adding Stable Lyse and Stable Store reagents from their respective reservoir ampules within each cartridge. Finally, the cartridges were transferred and stored in a −80°C freezer.

Automated stimulation and fixation of whole blood samples were done using a cartridge-based automated stimulation system (Fig. 2). This was designed and developed as a cartridge that contained three chambers for sample stimulation, three chambers for the reagents and one chamber for sample loading. One cartridge corresponded to samples from one study participant. The loading chamber contained a needle that punctured the vacutainer tube such that the sample was maintained in a sterile condition. Using a syringe, 1 ml of the whole blood sample was dispensed into the loading chamber of the cartridge (Step 1). The cartridge was then placed onto an instrument which via an integrated rotary valve dispensed an equal volume of whole blood (250 μl) into each of the three stimulation chambers (Step 2 to 3). The lyophilized reagents within each chamber then stimulated the blood samples for a 6 h timed interval (Step 4), following which the SMART TUBE stabilizer and fixative buffers were sequentially added to the samples and each incubated for a timed interval (Step 5). Finally, the cartridge was taken off the instrument and placed in the -80°C freezer until the sample was ready for further testing (Step 6). For the study we had three conditions—no stimulation (UNSTIM), DENV, and CHIKV peptide stimulation which was done for 6 h. A limitation of the cartridge design was the inability to include a 4th condition, so no positive control such as PMA+ Ionomycin stimulation was included.

### Manual stimulation and fixation

Fresh whole blood samples were stimulated and fixed as previously described.^19^ Briefly, after blood draw, whole blood was divided into unstimulated, antigen stimulated, PMA+ Ionomycin stimulated, and lipopolysaccharide (LPS) stimulated samples. For each condition, 500 μl whole blood was stimulated with LPS, CHIKV and DENV antigens and lysates for 6 h and with PMA+

Ionomycin for 4 h. During incubation, Brefeldin A and Monensin were added to both unstimulated and stimulated samples. Post stimulation, whole blood samples were lysed for 15 min at RT using SMART TUBE Stable Lyse buffer, followed by fixation for 15 min at RT with SMART TUBE Stable Store buffer. Samples were then transferred to cryovials and stored in the −80°C freezer until processing.

### CyTOF

**Figure 3.**
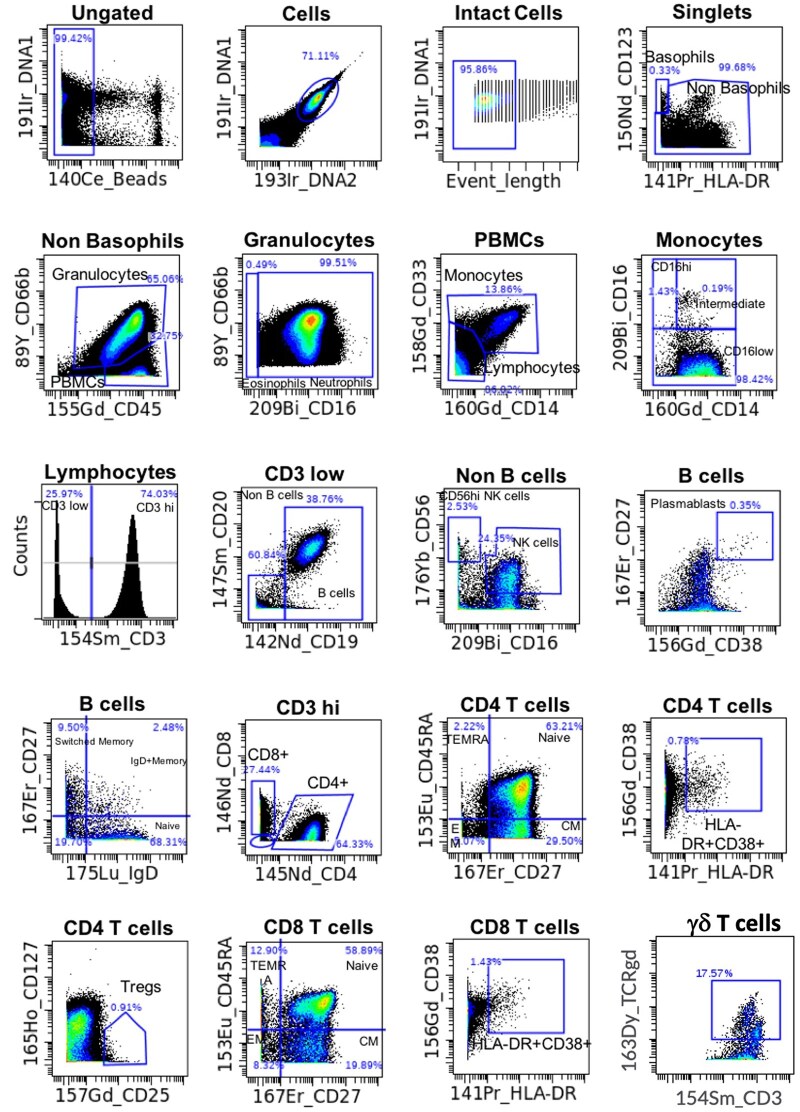
CyTOF gating schematic. After normalization using the MATLAB normalizer, the normalized.fcs files were uploaded to Cytobank (www.cytobank.org). Cells were gated to exclude normalization beads, debris, and event length parameters. From intact singlets, the major cell populations—including basophils, non-basophils, granulocytes, and PBMCs—were identified. Within the PBMC population, hierarchical gating was applied to further identify key cell populations, such as B cells, memory B cell subsets, plasmablasts, and CD4+ and CD8+ T cells and their memory subsets, Tregs, γδ T cells, NK cells, and monocytes.

Fixed whole blood samples from 81 participants as well as fixed whole blood control samples (unstimulated and PMA+ Ionomycin stimulated) were thawed in cold water for 15 min. After thawing, samples were transferred to a deep well plate, processed, barcoded and labeled for surface and intracellular markers (Table 1) as previously described.^19^ The control PMA+ Ionomycin stimulated fixed control sample was excluded from the barcode pool to eliminate potential false positive readouts due to possible contamination with the barcoded samples. Post-acquisition on CyTOF Helios (Standard BioTools), raw .fcs files were normalized using the MATLAB normalizer available on github.com (https://github.com/nolanlab/bead-normalization/releases) using a reference bead file. Normalized barcoded .fcs files were then debarcoded using the Standard BioTools software, prior to uploading to Cytobank (www.cytobank.org) for manual gating and analysis. As shown in Fig. 3, single intact cells were gated based on beads, DNA content and event length parameters. From intact singlets, the major cell populations including basophils, non-basophils, granulocytes and PBMCs were gated. From the PBMCs, hierarchically, the major cell populations such as B cells and memory B cell subsets, plasmablasts, CD4^+^ and CD8^+^ T cells with their respective memory subsets and activation status, Tregs, γδ T cells, NK cells and monocytes were identified.

**Table 1.** Comprehensive CyTOF staining panel for immune cell subset phenotyping and functional analysis, including metal labeling, specificity, antibody clone details, sources and staining.

Metal label	Specificity	Clone	Source	Staining
89Y	CD66b	G10F5, BioLegend	In-house	Surface
102Pd-110Pd	Barcode			
113In	CD57	HCD57, BioLegend	In-house	Surface
140Ce	Beads	n/a		
141Pr	HLA-DR	L243 (G46-6), BioLegend	In-house	Surface
142Nd	CD19	HIB19	Standard BioTools	Surface
143Nd	IL-10	JES3-9D7, BioLegend	In-house	Intracellular
144Nd	1L-4	MP4-25D2	Standard BioTools	Intracellular
145Nd	CD4	RPA-T4	Standard BioTools	Surface
146Nd	CD8	RPA-T8	Standard BioTools	Surface
147Sm	CD20	2H7	Standard BioTools	Surface
148Nd	CD40	5C3, BioLegend	In-house	Surface
149Sm	CD11c	Bu15, BioLegend	In-house	Surface
150Nd	CD123	6H6, BioLegend	In-house	Surface
151Eu	CD107a	H4A3	Standard BioTools	Intracellular
152Sm	TNF-α	Mab11	Standard BioTools	Intracellular
153Eu	CD45RA	HI100	Standard BioTools	Surface
154Sm	CD3	UCHT1	Standard BioTools	Surface
155Gd	CD45	HI30, BioLegend	In-house	Surface
156Gd	CD38	HB-7, BioLegend	In-house	Surface
157Gd	CD25	MA251, BioLegend	In-house	Surface
158Gd	CD33	P67.6, BioLegend	In-house	Surface
159Tb	GM-CSF	BVD2-21C11	Standard BioTools	Intracellular
160Gd	CD14	M5E2	Standard BioTools	Surface
161Dy	IFN-γ	4S.B3, eBioscience	In-house	Intracellular
162Dy	CD69	FN50	Standard BioTools	Intracellular
163Dy	TCR-γδ	B1, BioLegend	In-house	Surface
164Dy	IL-17	N49-653	Standard BioTools	Intracellular
165Ho	CD127	A019D5	Standard BioTools	Surface
166Er	IL-2	MQ1-17H12	Standard BioTools	Intracellular
167Er	CD27	L128	Standard BioTools	Surface
168Er	CD40L	24-31	Standard BioTools	Intracellular
169Tm	CCR7	150503, R&D Systems	In-house	Surface
170Er	PD1	EH12.1, BD Pharmingen	In-house	Surface
171Yb	Granzyme B	GB11	Standard BioTools	Intracellular
172Yb	FcRL5	509f6, BioLegend	In-house	Surface
173Yb	Perforin	B-D48, Abcam	In-house	Intracellular
174Yb	CD21	Bu32, BioLegend	In-house	Surface
175Lu	IgD	IA6-2, BioLegend	In-house	Surface
176Yb	CD56	NCAM16.2	Standard BioTools	Surface
191Ir	DNA1	n/a		
193Ir	DNA2	n/a		
209Bi	CD16	3G8	Standard BioTools	Surface

### CyTOF statistical analysis

Data shown are in the form of relative percentages of the parent cell population as derived from Cytobank gating. GraphPad Prism 8.0 (GraphPad Software, Inc., La Jolla, California, USA) was used to plot graphs. To compare cytokine differences between unstimulated and antigen stimulated conditions across the T cell populations (n = 79), we used the 2-way ANOVA (or mixed effects model) statistical analysis within the GraphPad Prism 8.0 software. Correction for multiple comparisons was performed using the Dunnett’s multiple comparisons test to generate adjusted *P*-values (*P < 0.05; **P < 0.01; ***P < 0.001; ****P < 0.0001). The error bars represent the mean with SEM. Samples from 2 participants were excluded from the analysis due to low PBMC counts.

### Confidence interval test

This test calculates the confidence interval around the difference between 2 proportions, that of cytokine-producing cells in response to peptide stimulation versus no stimulation. If the confidence interval overlaps with zero, the peptide response is not significantly above background. The CI test is independently calculated for each study participant.

### Flow cytometry

**Figure 4.**
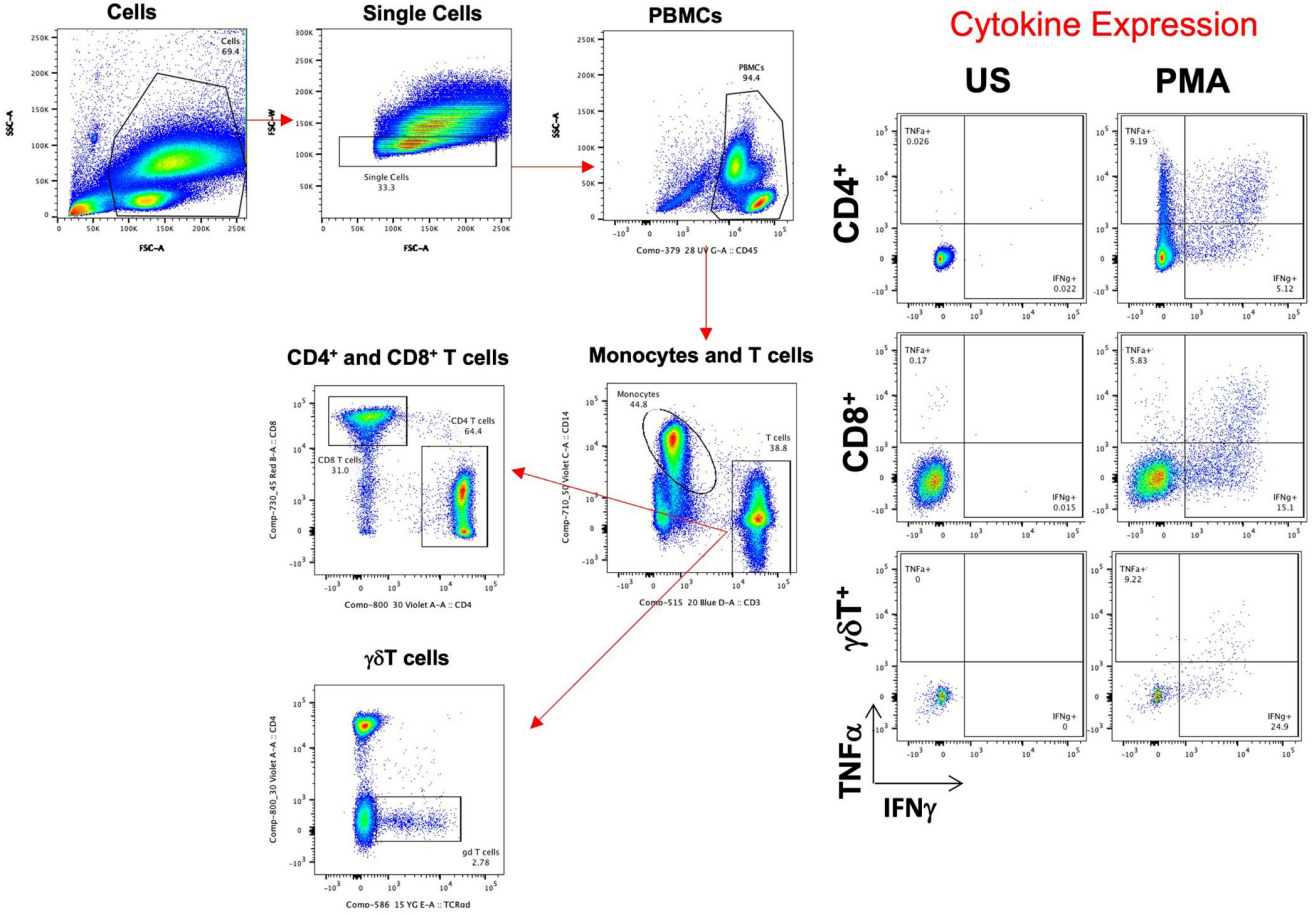
Flow cytometry gating schematic. FlowJo software v.10 (BD) was used to map the immune cell populations. Single intact cells were gated based on FSC-A, SSC-A, and FSC-W parameters. From these intact singlets, PBMCs were identified using SSC-A and the CD45 marker. Within the PBMC population, we sequentially identified lineage-specific cell populations, including monocytes, B cells, NK cells, and T cells, with subsets of CD4+ T cells, CD8+ T cells, and γδ T cells.

Antigen-specific T cell responses on forty participants were measured using a targeted intracellular cytokine panel. Fixed frozen whole blood cartridges for a given participant and control samples were thawed for 15 min in cold water. After thawing, samples were transferred to a deep-well plate. Additional lysis was performed using erythrocyte lysis buffer if red blood cells were observed post thawing. Samples were washed twice with FACS buffer (PBS + 5% FBS) by centrifuging at 974 × *g* for 8 min at RT. The cells were then immunolabeled with the surface antibody cocktail (Table 2) for 30 min in the dark at RT. Subsequently, cells were washed twice with FACS buffer followed by additional fixation with 1× BD FACS Lysing solution for 15 min at RT in the dark. After fixation, cells were washed twice with 1× permeabilization buffer (BD Biosciences) at 974 × *g* for 8 min at RT. The permeabilized cells were then incubated with the intracellular cytokine antibody cocktail (Table 2) for 30 min on ice, in the dark. Cells were then washed twice with FACS buffer at 974 *x g* for 8 min at 4°C. Finally, cells were fixed in 2% PFA and kept overnight at 4°C. The next day, cells were acquired on a BD Symphony X50 Flow Cytometer and the associated BD FACS Diva software. FlowJo software v.10 (BD) was used to analyze the flow cytometry data. As seen in Fig. 4, single intact cells were gated based on FSC-A, SSC-A and FSC-W parameters. From the intact singlets, PBMCs were gated using the SSC-A and CD45 marker. From the PBMCS, we sequentially identified the lineage cell populations such as the Monocytes, B cells, NK cells, T cells including CD4^+^ T cells, CD8^+^ T cells and γδ T cells.

**Table 2.** Flow Cytometry ICS targeted T cell panel detailing antibody fluorochrome, channel, clone, sources and staining.

Fluorochrome	Channel	Antibody	Clone	Vendor	Staining	**Vol. per reaction (**μl**)**
Alexa Fluor 488	Blue D	CD3	UCHT1	BIOLEGEND	Surface	5
Alexa Fluor 647	Red C	TNFα	Mab11	BIOLEGEND	ICS	5
APC-R700	Red B	CD8	RPA-T8	FISHER SCIENTIFIC	Surface	5
BV605	Violet E	CD16	3G8	BIOLEGEND	Surface	5
BV711	Violet C	CD14	M5E2	BIOLEGEND	Surface	5
BV785	Violet A	CD4	RPA-T4	BIOLEGEND	Surface	5
BUV395	UVG	CD45	HI30	FISHER SCIENTIFIC	Surface	5
PE	Y/G E	TCRγδ	B1	BIOLEGEND	Surface	3
PE-Cy7	Y/G A	IFNγ	4S.B3	BIOLEGEND	ICS	5
BV421	Violet I	PD-1	EH12.1	FISHER SCIENTIFIC	Surface	5

### Flow cytometry statistical analysis

The remaining 40 participant samples were tested by flow cytometry. However, samples from nineteen participants (19/40) were excluded from the analysis due to low PBMC counts. Data shown are in the form of relative percentages of the parent cell population as derived from gating in FlowJo. GraphPad Prism 8.0 was used to plot graphs. To compare cytokine differences between unstimulated and antigen stimulated conditions across the T cell populations (n = 21), we used the Two-way ANOVA (or mixed effects model) statistical analysis within the GraphPad Prism 8.0 software. Correction for multiple comparisons was performed using the Dunnett’s multiple comparisons test to generate adjusted *P*-values (*P < 0.05; **P < 0.01; ***P < 0.001; ****P < 0.0001). The error bars represent the mean with SEM.

## Results

The goal of this study was to validate the performance of a novel easy-to-use miniaturized automated system to stimulate and store small volumes of whole blood samples (250 μl) that could be used later for functional assays. This study was conducted using a pediatric cohort exposed to DENV and CHIKV in Kenya. We performed Mass Cytometry (CyTOF) to profile fixed whole blood samples and have a comprehensive understanding of their phenotypic and functional immune changes in response to DENV and CHIKV by *ex vivo* peptide stimulation using the novel automated system. Fixed whole blood samples from 81 to 121 study participants were processed for CyTOF. Samples from two participants were excluded from CyTOF analysis due to low PBMC counts (n = 79). Our comprehensive functional analysis on the samples from 79 children revealed changes in cytokine expression limited to TNF-α and IFN-γ in the CD8^+^ T cells and γδ T cells.

### TNF-α^+^ and IFN-γ^+^ T cell responses to CHIKV and DENV antigens

**Figure 5.**
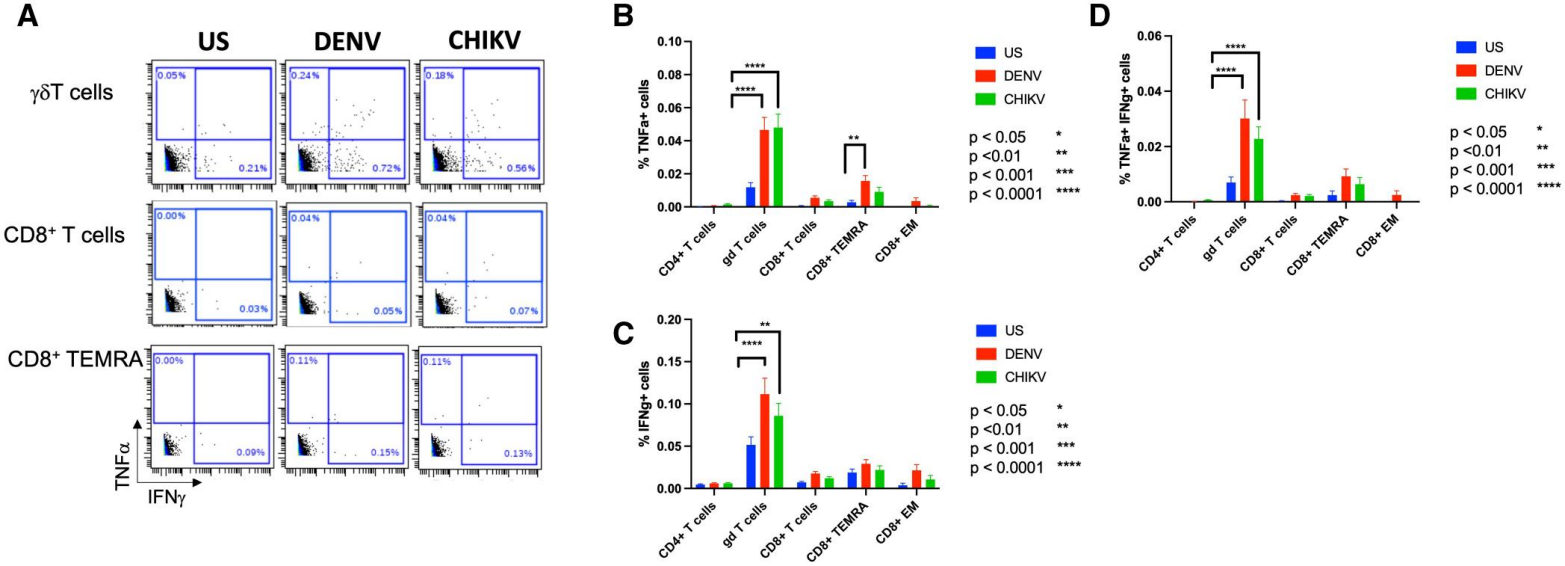
TNF-α and IFN-γ responses in T cells as determined by CyTOF. Representative dot plots showing cell frequencies of TNF-α and IFN-γ in response to DENV and CHIKV peptide stimulation in (A) γδ T cells (top panels), CD8+ T cells (center panels) and CD8+ TEMRA cells (bottom panels). Two-way ANOVA showed significant increase in the cell frequencies of (B) TNF-α +  γδ T cells and CD8+ TEMRA cells to DENV peptide and TNF-α^+^γδ T cells to CHIKV peptide. Frequencies of (C, D) IFN-γ+ and double positive TNF-α+ IFN-γ +  γδ T cells were significantly increased in response to both DENV and CHIKV peptides. Data is represented as a percentage of the parent population. **P < 0.01, ****P < 0.0001 (n = 79).

We comprehensively analyzed the functional responses to CHIKV and DENV peptide mix stimulation. We observed significant functional differences only in the T cell immune subsets. Specifically, our analysis showed only TNF-α^+^ and IFN-γ^+^ cytokine responses by the T cells. As seen in the representative dot plot, upon DENV and CHIKV antigen stimulation, an increase in the cell frequencies of TNF-α^+^ and IFN-γ^+^ γδ T cells, CD8^+^ T cells and CD8^+^ TEMRA memory cells were observed (Fig. 5A). Two-way ANOVA confirmed significant increases in the expression of TNF-α (Fig. 5B), IFN-γ (Fig. 5C) and double positive of TNF-α+IFN-γ+ (Fig. 5D) by the γδ T cells to both DENV and CHIKV antigens at the group level. Further, we performed the 95% confidence interval test at an individual level that showed ∼60% participants with increased γδ T^+^ TNF-α and IFN-γ responses to both antigen stimuli (Table 3). We also observed significant increases in the TNF-α^+^ responses by the CD8^+^ TEMRA subset (Fig. 5B) by 2-way ANOVA. For the CD8^+^ T cells there were no significant changes in the expression of TNF-α and IFN-γ to either antigen stimuli at the group level analysis (Fig. 5B, C). However, when we performed the 95% confidence interval test, we observed CD8^+^ TNF-α and IFN-γ responses to DENV and CHIKV antigens in a subset of participants (< 50%, Table 3). No significant responses to either antigen was observed in CD4+ T cells.

**Table 3.** 95% confidence interval above 0 for cytokine expression tested by CyTOF.

Number of participants showing significant responses	DENV	CHIKV
γδ T IFN-γ responses	53/79 = 67%	45/79 = 57 %
γδ T TNF-α responses	49/79 = 62%	47/79 = 60%
CD8^+^ IFN-γ responses	39/79 = 49%	35/79 = 44%
CD8^+^ TNF-α responses	26/79 = 33%	15/79 = 19%

As our CyTOF findings showed only TNF-α^+^ and IFN-γ^+^ T cell responses, we decided to validate the performance of the automated system using an alternative approach by flow cytometry using a more targeted T cell panel. We tested samples from the remaining study participants (n = 40/121) by flow cytometry. However, samples from nineteen (19 to 40) participants were excluded from the analysis due to low PBMC counts.

**Figure 6.**
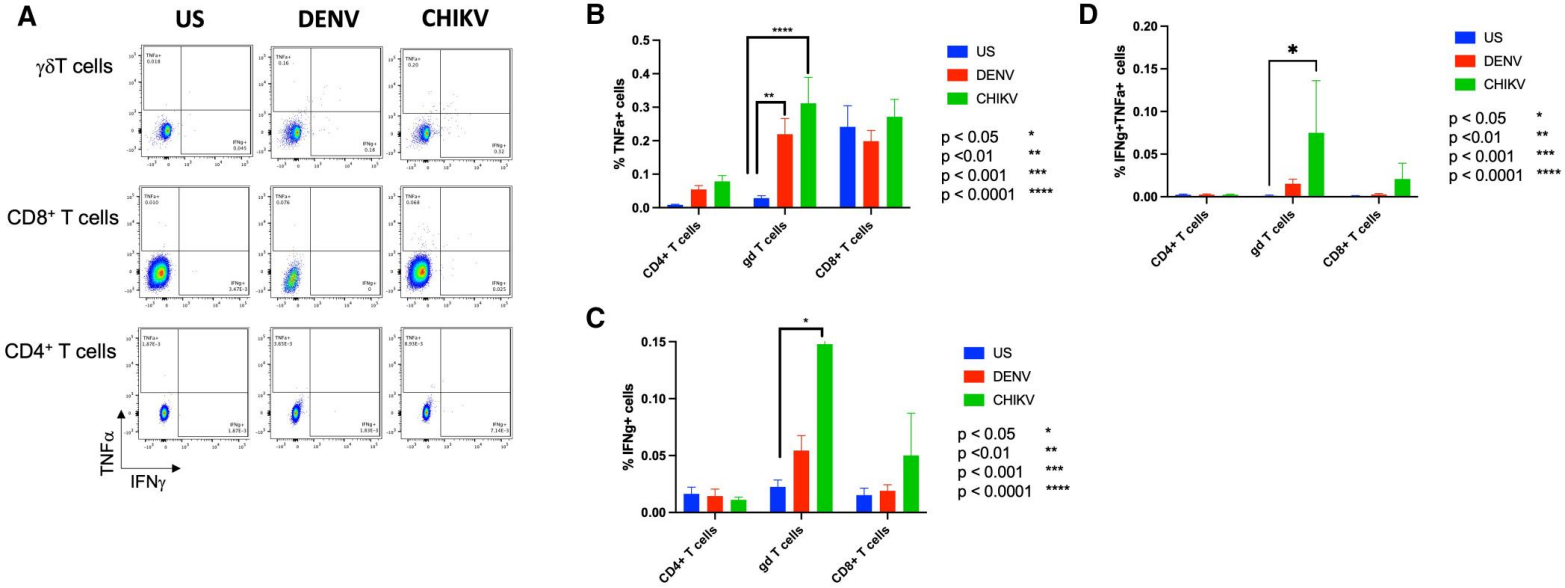
TNF-α and IFN-γ responses as determined by flow cytometry. Representative dot plots showing cell frequencies of TNF-α and IFN-γ in response to DENV and CHIKV peptide stimulation (A) γδ T cells (top panel), CD8+ T cells (center panel) and CD4+ T cells (bottom panel). Two-way ANOVA showed significant increase in the cell frequencies of (B) TNF-α^+^ (γδ T cells) to DENV and CHIKV peptides. Expression of (C, D) IFN-γ+ and double positive TNF-α+ IFN-γ+ were significantly increased in response to CHIKV peptide stimuli. Data is represented as a percentage of the parent population. *P < 0.05, **P < 0.01, ****P < 0.0001 (n = 21).

As seen in the representative dot plot, upon DENV and CHIKV antigen stimulation, an increase in the cell frequencies of TNF-α^+^ and IFN-γ^+^ were mostly observed in the γδ T cells compared to the CD4^+^ T cells and CD8^+^ T cells (Fig. 6A). Two-way ANOVA confirmed that γδ T+ TNFα expression was significantly increased to both DENV and CHIKV peptide stimuli (Fig. 6B), validating our findings from CyTOF. IFN-γ and double positive of TNF-α+IFN-γ+ expression was also increased in response to CHIKV peptide stimulation in γδ T cells (Fig. 6C, D). Additionally, we performed the 95% confidence interval test at the individual level that showed 10 -15% participants with increased γδ T^+^ TNF-α and IFN-γ responses to both antigen stimuli (Table 4). CD8^+^ TNF-α and IFN-γ responses to DENV and CHIKV antigens were also observed in 10-30% of participants at the individual level but not at the group level (Table 4).

**Table 4.** 95% confidence interval above 0 for cytokine expression tested by flow cytometry.

Number of participants showing significant responses	DENV	CHIKV
γδ T IFN-γ responses	3/21 = 14%	2/21 = 10%
γδ T TNF-α responses	2/21 = 10%	3/21 = 14%
CD8^+^ IFN-γ responses	4/21 = 19%	3/21 = 14%
CD8^+^ TNF-α responses	2/21 = 10%	6/21 = 29%

Stimulated and fixed whole blood samples by the novel miniaturized automated cartridge system showed increases in TNF-α and IFN-γ expression by the γδ T cells to antigen stimuli by two independent testing approaches—CyTOF and flow cytometry. As the cytokine responses to antigen stimuli were of a lower magnitude, and a limitation of the automated prototype design was the inability to include a “positive control” chamber, we therefore decided to test samples from a subset of the study participants by stimulating and fixing their whole blood samples manually. With this approach along with stimulating whole blood samples with DENV and CHIKV peptides, we stimulated samples with LPS and PMA+ionomycin as positive controls.

### Cytokine responses by automated versus manual stimulation

Samples from four study participants for whom we had processed samples using the automated system, were manually stimulated by the DENV and CHIKV antigens, LPS and PMA+ionomycin, followed by fixation. The dot plots show TNF-α and IFN-γ expression by the γδ T cells in the automated system (Smart Tube) and manual stimulation, for each of the 4 participants (Fig. S3). Each row corresponds to a single participant and each column corresponds to the stimulation condition. The dot plots show low level γδ T cell antigen responses by manual stimulation as was observed with the automated stimulation system, though the occurrence and magnitude of these responses was not highly correlated.

### ELISA and T cell assay comparisons

**Figure 7.**
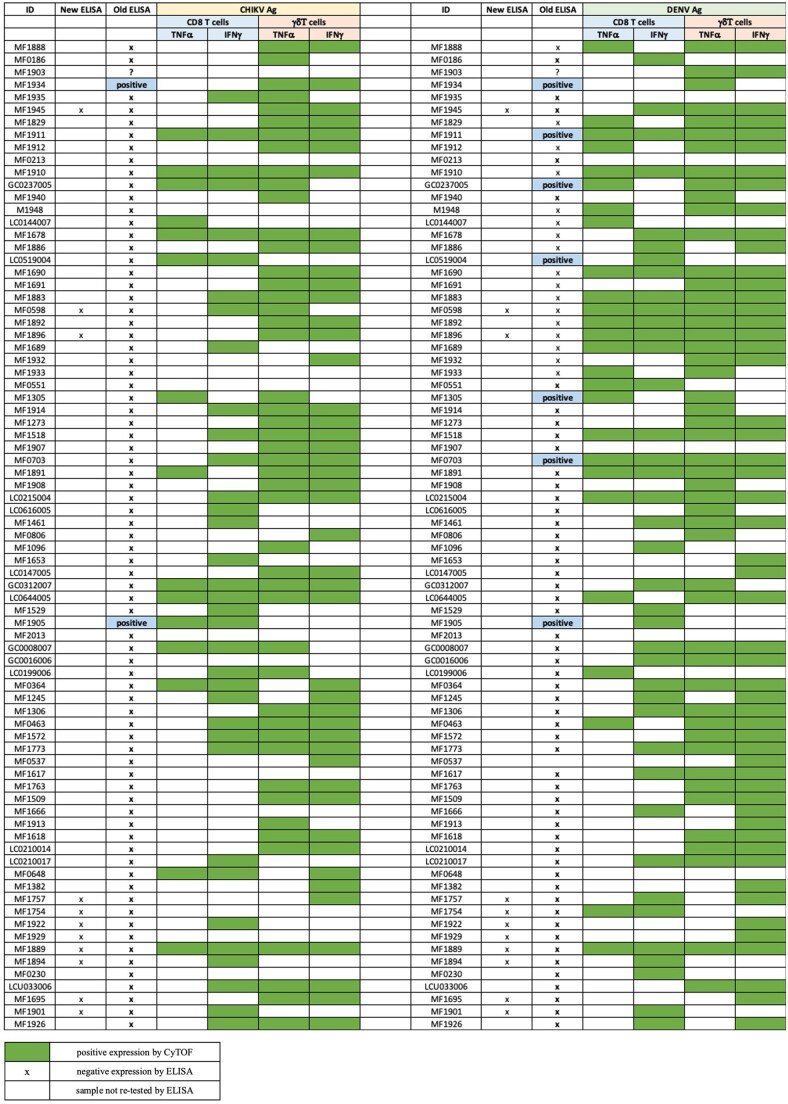
Comparisons of ELISA and confidence interval results from CyTOF. Comparison of serology ELISA results with confidence intervals from CyTOF was conducted to evaluate the correlation between seropositive responses by ELISA and T cell responses to CHIKV and DENV antigens as measured by CyTOF.

**Figure 8.**
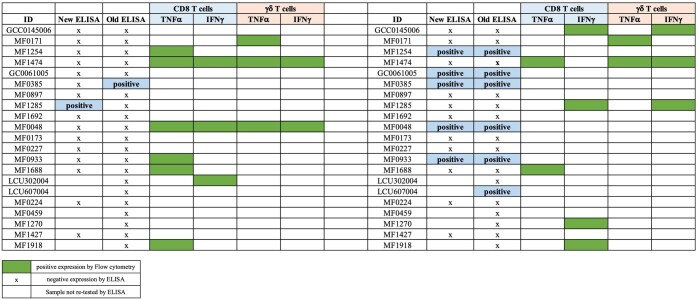
Comparisons of ELISA and confidence interval results from flow cytometry. Comparison of serology ELISA results with confidence intervals from Flow cytometry was conducted to evaluate the correlation between seropositive responses by ELISA and T cell responses to CHIKV and DENV antigens as measured by Flow cytometry.

Additionally, we also performed comparisons between the serology ELISA and T cell assays—CyTOF and flow cytometry. Neither CyTOF nor flow cytometry responses correlated well with ELISA serology. CyTOF in particular showed a higher proportion of participants with T cell responses to either or both CHIKV and DENV antigens, compared to the proportion seropositive by ELISA. The ELISA testing was repeated on a subset of individuals (mostly those also tested by flow cytometry) and largely recapitulated the earlier results, confirming the discordance with the cellular assays (Figs 7 and 8).

## Discussion

In this study, we used an innovative automated whole blood cellular analysis system along with CyTOF single-cell high dimensional technology to study immune responses in young children from Msambweni, Kenya, exposed to CHIKV and/or DENV viruses. The broad dimensionality provided by CyTOF enabled us to thoroughly investigate immune responses to DENV and CHIKV antigen stimuli.

Our mass cytometry analysis of whole blood from the pediatric cohort revealed γδ T^+^ TNF-α and IFN-γ responses following both antigen stimuli. Studies in mice have recognized γδ T cells’ as important players in protective immunity both in recruiting crucial inflammatory cell populations and mitigating tissue damage caused by oxidative stress particularly during the early responses to CHIKV.^20^^,^^21^ Furthermore, a study by Chen-Yu Tsai et al. has demonstrated that γδ T cells isolated from PBMCs of individuals with acute DENV infection contribute to the immune response against DENV virus by serving as an early source of IFN-γ and can eliminate the DENV virus-infected cells by the upregulation of immune markers involved in activation, proliferation, and degranulation.^22^ In our study, blood samples were drawn from well children without acute infections who had a prior history of arbovirus exposure to understand memory responses. Therefore, our findings of γδ T^+^ TNF-α and IFN-γ responses to both antigens suggests that γδ T cells may play an important role in protective immunity in this pediatric cohort. Further investigation to comprehensively understand the mechanics of immune protection by the γδ T cells would aid significantly in developing preventive and therapeutic interventions.

Previous studies have also highlighted the dual protective and pathogenic effects of α/β T cells in the context of DENV infection.^23^ In the case of DENV, the protective role of CD8 T cells involves elimination of infected cells and the production of proinflammatory cytokines like IFN-γ.^24^^,^^25^ Furthermore, human T cells producing TNF-α and IFN-γ specific to DENV virus have been documented.^26^^,^^27^ A transcriptome study by Tian et al., revealed that DENV-specific CD8+ T cell subsets, particularly TEMRA cells to be fully activated and polyfunctional with limited transcriptional responses.^28^ Many studies have also shown CD8+ TEMRA cells to play a protective role against several viral pathogens including HIV, cytomegalovirus (CMV), Epstein-Barr virus (EBV), influenza and yellow fever in humans.^29–32^ Within our mass cytometry data, we observed a notable increase in TNF-α positive cells within CD8 TEMRA subsets in response to DENV stimuli. Notably, our analysis did not reveal the induction of other cytokines by CD8+ T cells or their memory subsets in response to DENV or CHIKV stimuli. It has also been reported that in CHIKV and DENV infection, CD8 T cells are activated during the acute phase of infection whereas CD4 T cells get activated during the chronic stages of infection where they promote B cells and CD8 T cell responses. Moreover, during the advanced phases of DENV infection, CD4 T cells play a crucial role in generating memory T cells by targeting the virus’ structural proteins.^24^^,^^33^ In our study, we did not observe cytokine productions by CD4+ T cells in response to both CHIKV and DENV antigens.

As the antigen-stimulated responses were of a low magnitude, we independently performed flow cytometry experiments on a subset of the cohort, employing a targeted panel. Our findings from the flow cytometry data supported the CyTOF results. The frequency of γδ T^+^ TNF-α+ expressing cells was significantly increased to DENV and CHIKV antigen stimuli. Surprisingly, our flow cytometry analysis revealed significant IFN-γ expression by the γδ T cells only in response to CHIKV. It is possible that this feature of IFN-γ expression relates to the generally low severity of DENV infection in Kenya.^12^

Furthermore, we processed samples by manual stimulation from a subset of participants that were previously processed by the automated prototype. However, of note, the samples for the manual stimulation were recruited and processed from the participants almost a year after sample collection for stimulation by the automated system. It is therefore not surprising that we did not observe a stronger correlation of cytokine responses to DENV and CHIKV peptide stimuli. However, there were at least hints of similar responses with manual stimulation. Furthermore, in the presence of LPS and PMA+ionomycin, we observed a high cytokine response, confirming the samples were viable and functional.

Finally, we also compared findings from our independent T cell assays with ELISA serology test findings. For the most part, we found a discordance in the results between these two assays. We hypothesize at least two major reasons for this discrepancy: first, the sensitivity and specificity of the two types of assays could be quite different. While we used specific peptides in the T cell assay, we cannot rule out some level of cross-reactivity with other unknown antigens. Conversely, the ELISA assay may miss low-level positives or those who respond to different antigens than the ones included in the test. A second major reason could be biological discordance not everyone with a cellular response might produce a detectable antibody response, and vice versa.^34–42^ Also, not all antigens were tested in the cellular assay, so responses may have been missed. Additionally, IgG levels could wane over time resulting in missed antibody responses.

While we made every effort to develop and deploy this innovative automated prototype to extend our research into remote areas, we acknowledge several limitations. These include the absence of a positive control chamber in the prototype design, limited data on whether the peptide mixes used would elicit significant immune responses, lack of confirmation of infection history and the current unavailability of a commercial version of the system.

In conclusion, our study highlights the power of using automated and single-cell technologies in research to reveal crucial insights. Related to this, our findings suggest that the novel automated whole blood cellular analysis system has the potential to enable detailed immune function evaluation, particularly in pediatric populations with limited sample volumes. The combination of automated blood stimulation and stabilization with the powerful CyTOF technology can be extended to research on other viral and infectious diseases. This would allow for improved understanding of cellular immune responses in pediatric populations. Such insights could be valuable for the development of vaccines and effective treatments leading to improved healthcare and disease management.

## Supplementary Material

vlaf006_Supplementary_Data

## Data Availability

Data available on request.
