# Correction to: Comprehensive immune profiling of dengue and chikungunya viral responses using a novel miniaturized automated whole blood cellular analysis system and mass cytometry in a pediatric cohort in Msambweni, Kenya

**DOI:** 10.1093/immhor/vlaf020

**Published:** 2025-04-27

This is a **correction** to:

Sangeeta Kowli, Amy Krystosik, Matthew Hale, Francis Mutuku, Jael S Amugongo, Said L Malumbo, Phillip K Chebii, Priscillah W Maina, Kavita Mathi, Elysse N Grossi-Soyster, Mary Rieck, Angelle Desiree LaBeaud, and Holden T Maecker. Comprehensive immune profiling of dengue and chikungunya viral responses using a novel miniaturized automated whole blood cellular analysis system and mass cytometry in a pediatric cohort in Msambweni, Kenya. ImmunoHorizons. 9:vlaf006 https://doi.org/10.1093/immhor/vlaf006

In the originally published version of this manuscript, The source of the DENV peptides was incorrect.

The first sentence under the “DENV and CHIKV peptide selection” heading in the Materials and Methods:

“An equimolar mega pool mix of 268 peptides (9 to 15 amino acids for each peptide) corresponding to the known epitopes of DENV were obtained from Dr Alexander Sette at La Jolla Institute for Allergy and Immunology (San Diego, California, USA).”

Should be: “An equimolar mega pool mix of 268 peptides (9 to 15 amino acids for each peptide) corresponding to the known epitopes of DENV were obtained from Dr. Alessandro Sette and Dr. Daniela Weiskopf at La Jolla Institute for Immunology.”

In the Acknowledgments, the second sentence:

“The authors thank Dr Alexander Sette at La Jolla Institute for Allergy and Immunology (San Diego, California, USA) for providing them with the DENV peptide megapool.”

Should be: “The authors thank Dr. Alessandro Sette and Dr. Daniela Weiskopf at La Jolla Institute for Immunology (San Diego, California, USA) for providing them with the DENV peptide megapool.”

This error has been corrected.

