# Supplementary material for: Comprehensive immune profiling of dengue and chikungunya viral responses using a novel miniaturized automated whole blood cellular analysis system and mass cytometry in a pediatric cohort in Msambweni, Kenya
Source: Immunohorizons. 2025 Mar 6;9(4):vlaf006. doi: 10.1093/immhor/vlaf006 (PMC11884800; doi:10.1093/immhor/vlaf006)
Supplement: vlaf006_Supplementary_Data [file vlaf006_supplementary_data.zip › Kowli et al_Supplemental Data.docx]

**SUPPLEMENTARY FIGURES:**


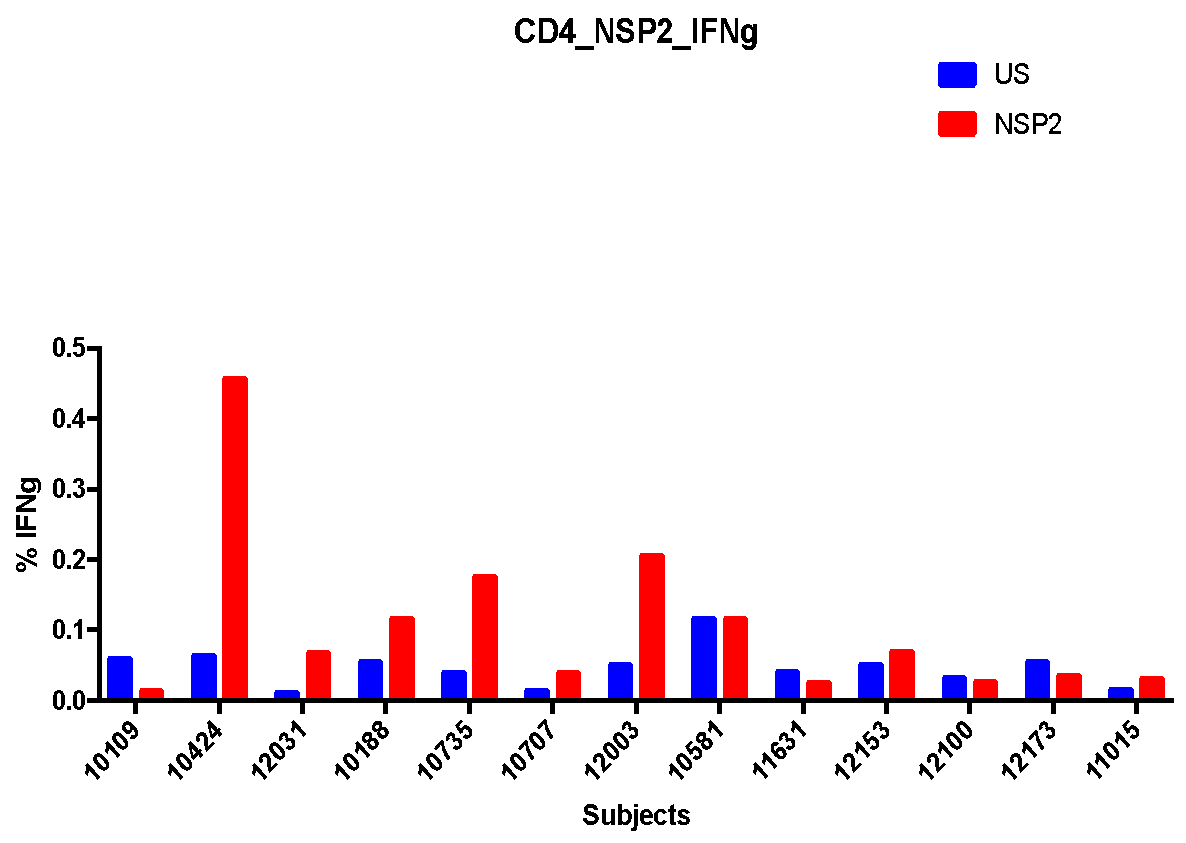


**Figure S1: CHIKV NSP2 CD4^+^ IFNγ responses.** PBMCs from healthy donors (n = 13) exhibited the strongest CD4+ IFNγ response following a 6-hour stimulation with CHIKV NSP2 peptide.

**Figure S2: Lyosphere Selection for Protein Inhibitors Brefeldin A and Monensin.** PBMCs from a healthy donor (n=1) were tested under unstimulated (US) and stimulated (PMA/ionomycin) conditions with three lyosphere formulations—B144, B346, and B407—using CyTOF. Each formulation included brefeldin A (BFA) and/or monensin (Mon). Controls included brefeldin A and monensin prepared in TBA and PBS. B407 was selected for its lowest background signal while preserving CD4+ and CD8+ T cell cytokine responses, specifically IFNγ, TNFα, IL-2, IL-4, and IL-17.

**
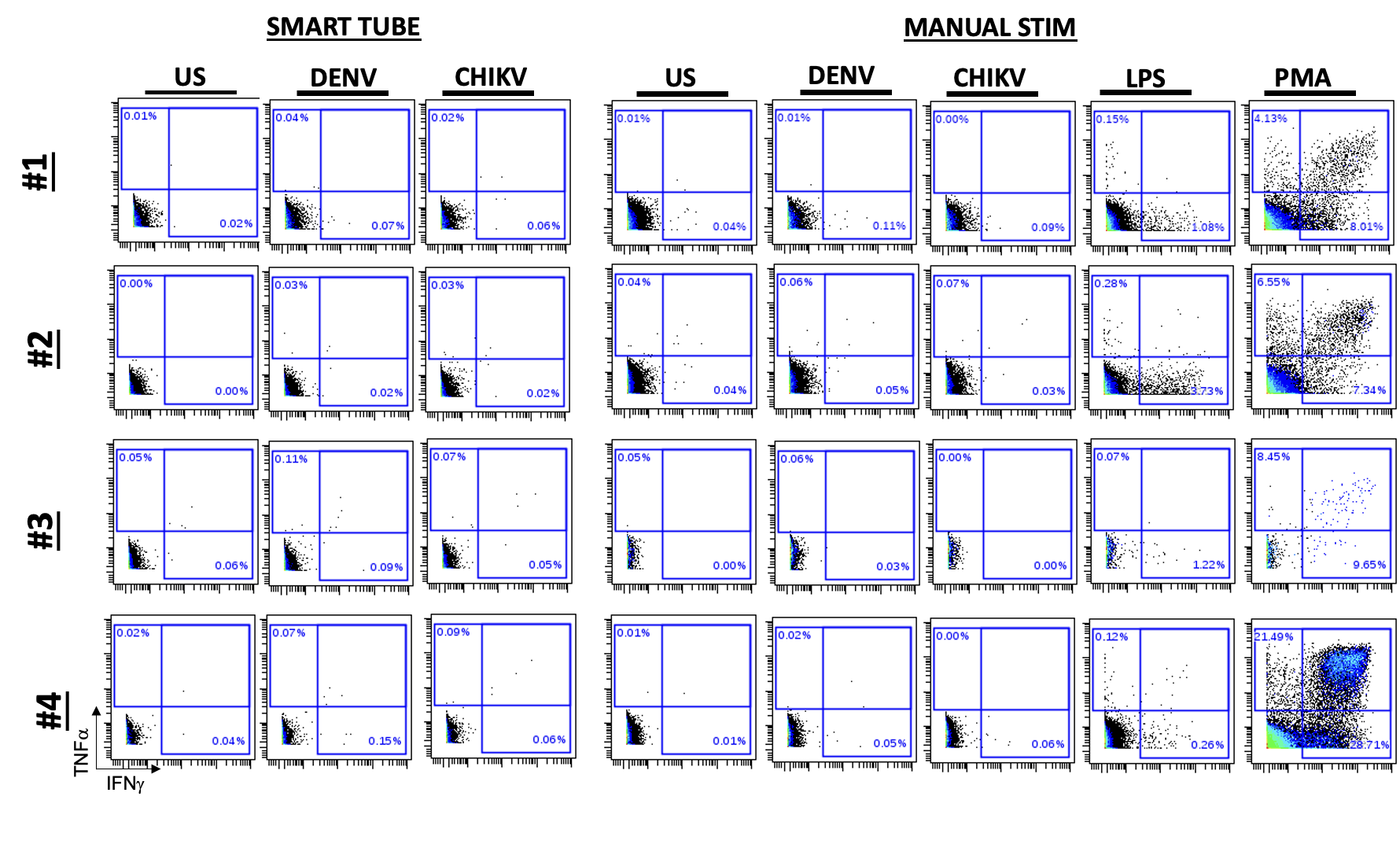
**

**Figure S3: Cytokine responses by automated versus manual stimulation.** Comparisons of TNFα and IFNγ expression by γδT cells were conducted across samples from four participants (corresponding to each row) and different stimulation conditions (each column) using both the automated prototype and manual stimulation methods. Low-level γδT cell antigen responses were observed in both stimulation methods; however, the occurrence and magnitude of these responses showed low correlation between the two approaches.
